# The Personality Trait of Intolerance to Uncertainty Affects Behavior in a Novel Computer-Based Conditioned Place Preference Task

**DOI:** 10.3389/fpsyg.2016.01175

**Published:** 2016-08-09

**Authors:** Milen L. Radell, Catherine E. Myers, Kevin D. Beck, Ahmed A. Moustafa, Michael Todd Allen

**Affiliations:** ^1^Department of Veterans Affairs, Veterans Affairs New Jersey Health Care System, East OrangeNJ, USA; ^2^Department of Pharmacology, Physiology and Neuroscience, New Jersey Medical School, Rutgers University, NewarkNJ, USA; ^3^School of Social Sciences and Psychology and Marcs Institute for Brain and Behaviour, University of Western Sydney, SydneyNSW, Australia; ^4^School of Psychological Sciences, University of Northern Colorado, GreeleyCO, USA

**Keywords:** uncertainty, decision making, conditioned place preference (CPP), personality, addiction, humans

## Abstract

Recent work has found that personality factors that confer vulnerability to addiction can also affect learning and economic decision making. One personality trait which has been implicated in vulnerability to addiction is intolerance to uncertainty (IU), i.e., a preference for familiar over unknown (possibly better) options. In animals, the motivation to obtain drugs is often assessed through conditioned place preference (CPP), which compares preference for contexts where drug reward was previously received. It is an open question whether participants with high IU also show heightened preference for previously rewarded contexts. To address this question, we developed a novel computer-based CPP task for humans in which participants guide an avatar through a paradigm in which one room contains frequent reward (i.e., rich) and one contains less frequent reward (i.e., poor). Following exposure to both contexts, subjects are assessed for preference to enter the previously rich and previously poor room. Individuals with low IU showed little bias to enter the previously rich room first, and instead entered both rooms at about the same rate which may indicate a foraging behavior. By contrast, those with high IU showed a strong bias to enter the previously rich room first. This suggests an increased tendency to chase reward in the intolerant group, consistent with previously observed behavior in opioid-addicted individuals. Thus, the personality factor of high IU may produce a pre-existing cognitive bias that provides a mechanism to promote decision-making processes that increase vulnerability to addiction.

## Introduction

Some individuals exposed to drugs of abuse develop addiction while others do not. One factor mediating this difference in outcomes may be personality traits that confer biases in decision making, such as a tendency to pursue familiar sources of reward at the expense of exploring other (possibly more rewarding) options. Such individual differences have been studied in the context of anxiety, but some of the same personality traits may also confer vulnerability to addiction. Addiction has a high comorbidity rate with anxiety disorders (Merikangas et al., 1998; Grant et al., 2004). Based on their comorbidity, it is not surprising that both types of disorders share other common features, including behaviors such as withdrawal or avoidance, changes in learning, and maladaptive decision making (e.g., risk taking, chasing reward). This alteration in decision making is not limited to decisions about drugs, but can also affect reward in general (Clark and Robbins, 2002). Drug use continues regardless of the negative consequences (e.g., to health, income, family), as do anxiety behaviors. Addiction and anxiety also share some common neural mechanisms. Both come about through some form of associative learning to a maladaptive stimulus, specifically, anxiety via altered associative learning in the amygdala (Packard and Cahill, 2001; Packard, 2009) and addiction through altered reward learning in the mesolimbic dopamine system (Robinson and Berridge, 2001; Everitt and Robbins, 2005; Volkow et al., 2010). In addition, stress and anxiety can lead to increased drug use and relapse (Jacobsen et al., 2001; Sinha, 2001).

### Learning, Personality and Vulnerability

Some recent work has examined the effects of personality on vulnerability for anxiety disorders, and to a lesser extent, addiction. Overall, the results suggest that personality factors, including behavioral inhibition (BI) and harm avoidance, hypothesized to be risk factors for anxiety disorders, are associated with enhanced learning in a variety of tasks (Sheynin et al., 2013, 2014; Allen et al., 2014; Holloway et al., 2014). For example, BI is a temperamental tendency to withdraw from or avoid novel social and non-social situations (Kagan et al., 1987; Morgan, 2006). In addition to avoidance, BI includes social reticence and enhanced reactivity to novelty, threat, and uncertainty (Hirshfeld et al., 1992; Schwartz et al., 2003). BI has long been considered a vulnerability factor for the development of anxiety-related disorders including post-traumatic stress disorder (Myers et al., 2012; Clauss et al., 2015). Behaviorally inhibited individuals exhibit enhanced associative learning as measured by eyeblink conditioning with a tone conditioned stimulus (CS) and a corneal air puff unconditioned stimulus (US) (Allen et al., 2014, 2016; Holloway et al., 2014), and with increased avoidance in a computer-based task (Sheynin et al., 2014). Enhanced avoidance learning was also observed in male, but not female, opioid addicts undergoing methadone maintenance therapy, when compared to controls, using the same task (Sheynin et al., 2016).

In addition to these findings with basic classical conditioning and avoidance learning, the effects of personality factors have been examined with computer-based tasks involving economic decision making. For example, Radell et al. (2016) used a cognitive economic decision making task based on socials interactions (i.e., the trust game) with behaviorally inhibited individuals. This task, based on the version used by Delgado et al. (2005), had participants read the biographies of partners in the game that portrayed them as morally trustworthy (“good partner”), untrustworthy (“bad partner”), or neutral (“neutral partner”). On each trial, participants were shown a partner and were given a choice of keeping $1 or sharing $3 with that partner. If the money was shared, the partner had the choice of keeping it all or reciprocating by returning half of the money ($1.50). On any trial in which the participant chose to share, the partner always reciprocated with 50% probability, irrespective of how they were portrayed in the biography. Inhibited individuals tended to share with the neutral partner less than uninhibited individuals; however, this behavioral difference was not evident in the ratings of trustworthiness for the “neutral partner.” These results suggest that inhibited individuals may be predisposed to interpret neutral or ambiguous information more negatively, which may contribute to the tendency to avoid unfamiliar people characteristic of behaviorally inhibited temperament, and its relationship to anxiety disorders.

Probabilistic category learning tasks that include both reward and punishment trials have also revealed a role for anxiety vulnerability factors in economic decision making (Sheynin et al., 2013). On each trial, participants view a stimulus and are asked to categorize it. The categories are probabilistic in that each stimulus is a member of one category 80% of the time and a member of the other category 20% of the time. For some stimuli, correct categorization results in a reward (point gain) and incorrect categorization results in no feedback; for other stimuli, incorrect categorization results in a punishment (point loss) and correct categorization results in no feedback. Thus, performance on reward and punishment trials can be directly contrasted, as can the interpretation of the ambiguous “no-feedback” outcome, which can signal either failure to obtain reward or successful avoidance of punishment. Behaviorally inhibited individuals demonstrated better associative learning on both reward and punishment trials. Given the option to opt out of individual trials to avoid any chance of being punished or rewarded, inhibited individuals also preferred to opt out to avoid punishment (Sheynin et al., 2013). In a follow-up study, using this task, participants with severe symptoms of post-traumatic stress disorder exhibited enhanced learning, specifically on reward trials, relative to peers with fewer or no symptoms (Myers et al., 2013).

Extending this task to the topic of addiction, Myers et al. (2016) found that opioid-addicted individuals undergoing methadone maintenance therapy were more likely to abandon previous response rules and explore new alternatives when expectancies were violated (i.e., increased lose-shift behavior), relative to controls. Thus, addicted participants tended to respond based on immediate feedback, which may explain why they continue to pursue short-term reward while ignoring the long-term negative consequences of drug use (Myers et al., 2016). Likewise, in other decision-making tasks, addicts tend to choose small immediate rewards over larger delayed rewards, and display a number of other changes in decision making compared to control participants (Petry et al., 1998; Clark and Robbins, 2002). Additionally, at least some of these changes appear to persist even after long-term abstinence (Li et al., 2013). However, it is important to note that a preference for small immediate rewards over large delayed rewards is not specific to addicts – it has been shown in both humans and animals, and is a function of multiple factors including the length of the delay, age, intelligence (Mischel and Metzner, 1962), and the amount of reward (Green et al., 1997). Thus, addiction is only associated with an exaggeration of this preference, which may reflect increased impulsivity or reduced self-control (Madden et al., 2003).

### The Role of Uncertainty

One common feature of most of the tasks discussed above is some aspect of uncertainty that was associated with performance improvements. Acquisition of eyeblink conditioning was enhanced in anxiety-vulnerable individuals under protocols which included schedules of partial reinforcement with 50% CS alone and 50% US alone trials (Allen et al., 2014), and variability in trial timing (Allen et al., 2016). In contrast, vulnerability did not modulate performance on a standard 100% CS-US paired trials protocol. In the computer avoidance task (Sheynin et al., 2014), participants were given no instructions and had to learn, through trial-and-error, what behavior resulted in avoiding point loss. In the trust game (Radell et al., 2016), all partners shared 50% of the time regardless of the nature of their biographies but individuals with anxiety vulnerability only differed in how they treated the neutral partner. The probabilistic category learning task (Myers et al., 2016) involved uncertainty in that it was not possible to be correct 100% of the time based on the probabilistic nature of the categories. There was also a mix of reward and punishment trials, and no feedback was given on correct punishment trials and incorrect reward trials. Finally, tasks that pit immediate small rewards against larger delayed rewards (Petry et al., 1998; Clark and Robbins, 2002) may also involve perceived uncertainty in that there is no guarantee that the delayed reward will actually be received.

Given the possible role of uncertainty in most prior tasks examining the role of individual differences in anxiety and addiction vulnerability, the purpose of the current study was to test how personality can modulate economic decision making for rewards in healthy individuals, focusing on intolerance to uncertainty (IU) – another personality factor that has been linked to anxiety disorders (Dugas et al., 1997; Ladouceur et al., 1997; Birrell et al., 2011; Carleton, 2012; Grupe and Nitschke, 2013). IU can be defined as a tendency to perceive uncertain situations as aversive and stressful, and respond with BI and negative expectations about their possible consequences (Nelson et al., 2015). Initially, IU was linked to generalized anxiety disorder, and is a strong predictor of the tendency to worry (Dugas et al., 1997; Ladouceur et al., 1997). However, other studies suggest that it is not specific to that disorder, but constitutes a broader risk factor for the development and maintenance of anxiety and depression (Tolin et al., 2003; Carleton, 2012; Carleton et al., 2012).

In recent work, individuals undergoing treatment for opioid dependence had significantly higher IU, as measured with the IU scale (Carleton et al., 2007), compared to healthy controls, suggesting that IU may also be a risk factor in substance abuse and addiction. This evidence is, of course, correlational and a causal relationship, if any, remains to be established. Still, IU implies reduced risk taking, in contrast to substance abuse and addiction, associated with increased impulsivity and risk taking. Thus, if higher IU does contribute to addiction vulnerability, this relationship may be indirect and only appear in a subpopulation of individuals who, for example, may have started substance use as a form of self-medication for anxiety. Along the same lines, pathological gambling – also associated with increased risk taking – is also often comorbid with anxiety disorders (Lorains et al., 2011), which are, in contrast, linked to higher risk aversion (Maner and Schmidt, 2006) and greater IU (Ladouceur et al., 1997). As with the relationship between IU and addiction, these contradictory findings might be resolved if pathological gamblers are not a homogenous group of individuals, but rather consist of multiple subtypes, only one of which represents impulsive risk-takers (Blaszczynski and Nower, 2002). IU has also been linked to changes in economic decision making and reward system function (Nelson et al., 2015). Using a gambling task, Nelson et al. (2015) found IU could modulate event-related potential responses to gains and losses, which have been linked to activation in the ventral striatum and medial prefrontal cortex, and activation in the anterior cingulate cortex, respectively. Individuals with higher IU are more likely to perceive situations as uncertain, and have stronger emotional responses (e.g., increased anxiety) under those conditions (Ladouceur et al., 1997). They also tend to require additional information before making a decision, and paradoxically avoid cues that can lead to anxiety, which would in practice reduce the amount of information available for decision making (Ladouceur et al., 1997; Krain et al., 2006). Similar to drug addicted individuals, individuals with higher IU were more likely to choose small, low-probability rewards over larger but delayed high-probability rewards (Luhmann et al., 2011).

### Conditioned Place Preference

We sought to continue this line of research by investigating the role of IU on learning in a computer-based economic decision making task, similar to the conditioned place preference (CPP) paradigm widely applied to the study of addiction in animal models. CPP has been commonly used to measure the reward value of different drugs of abuse (for reviews, see Bardo and Bevins, 2000; Tzschentke, 2007). Here, drug-free subjects (typically rodents), are first allowed to explore an apparatus consisting of at least two distinct interconnected chambers to measure initial preference (i.e., by comparing time spent in each context). In subsequent conditioning sessions, the animal is injected with a drug and confined to one chamber. Similarly, the other context is paired with saline. Finally, drug-free subjects are once again allowed to choose between the compartments in order to assess preference. A large number of studies have shown animals spend more time in the drug-paired than in the saline-paired compartment for a wide variety of drugs, including opioids (e.g., heroin, methadone), and psychomotor stimulants (e.g., cocaine, amphetamine) (Bardo et al., 1995). CPP has also been observed for non-drug rewards including food (Spyraki et al., 1982), water, and access to sexual interaction (Oldenburger et al., 1992) or a running wheel (Lett et al., 2000).

Here, we report results from a computer-based CPP task where humans guide an avatar through a paradigm in which one room contains frequent reward and one contains less frequent reward. Following exposure to both contexts, participants were assessed for preference to enter the previously rich and previously poor room. IU was assessed via a self-report questionnaire. An important limitation of animal CPP as a model of human substance abuse and addiction is that rewards are simply administered by the experimenter, and are not contingent on behavior (e.g., animals are injected with drug or confined to a compartment containing reward). In contrast, humans choose to start taking the drug and control the frequency of administration. To address this concern, in the current task, obtaining reward was contingent on operant responding by the participants. We predicted that if IU contributes to decision making that can promote substance abuse and addiction, individuals with higher IU should show a stronger bias toward the previously rich room, compared to individuals with lower IU, who might be more prone to explore other options.

## Materials and Methods

### Participants

A total of 88 participants were recruited from the University of Northern Colorado, and received research credit in a psychology class as payment for their participation. Data from 12 participants were lost due to computer failure. The remaining sample (*n* = 76) contained 50 females and had a mean age of 20.7 (*SD* = 5.4, range = 18–56), and education of 13.8 years (*SD* = 1.4, range = 12–17). All participants provided informed consent before initiation of any behavioral testing. Procedures were approved by the Institutional Review Board at the University of Northern Colorado, and conformed to guidelines established by the Federal Government and the Declaration of Helsinki for the protection of human subjects.

### Procedure

**FIGURE 1:**
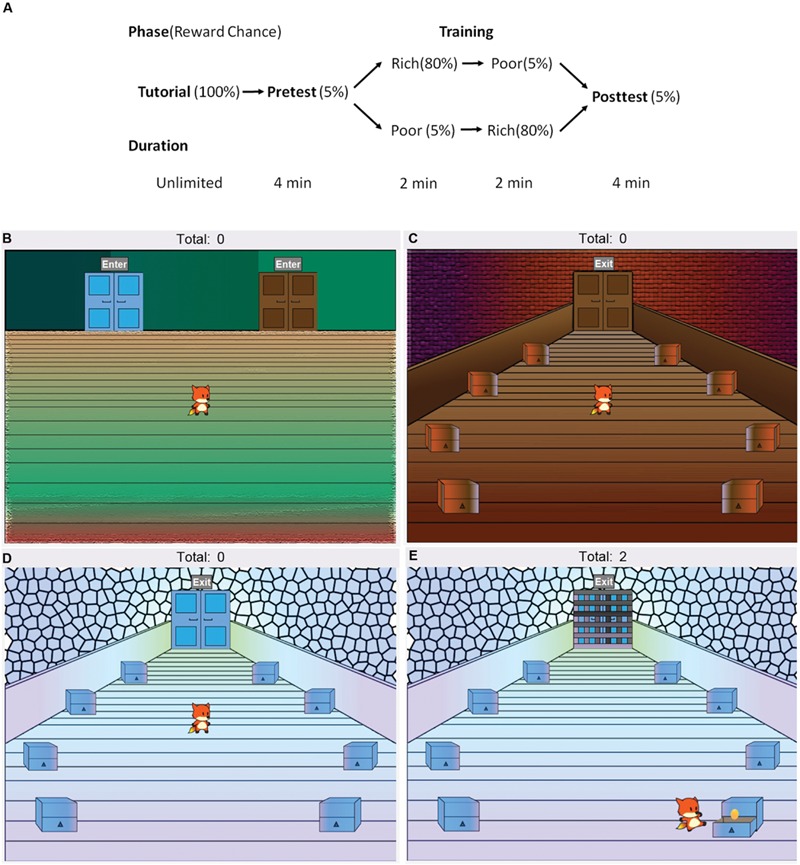
**Design of the computer-based conditioned place preference task. (A)** The task consisted of a tutorial, pretest, training and a posttest phase. **(B)** Participants controlled an avatar (the fox), which was placed in the lobby area (shown here) at the start of each phase. The lobby area contained two doors. During the pretest and posttest, participants were freely allowed to switch between a **(C)** blue and a **(D)** brown room by using the mouse to click on the doors, and could also click on the chests to search for golden eggs, increasing their total score. Each chest initially had a 5% chance of containing an egg. Whether the blue room door in the lobby was on the left or the right was counterbalanced. **(E)** In the training phase, participants were forced to enter one, then the other, room and locked inside. In one room (“rich room”), each chest initially had an 80% chance of containing an egg, in contrast to the other (“poor room”) where each chest initially had a 5% chance. Whether participants were forced to enter the rich or the poor room first during training was counterbalanced.

Testing took place in a quiet room. All participants completed the brief, 12-item version of the Intolerance to Uncertainty Scale (IUS-12; Carleton et al., 2007), and a computer-based CPP task programmed in the Java 8 language (Oracle Corporation, Redwood City, CA, USA), administered on a desktop computer running Windows. The task, illustrated in **Figure 1**, consisted of a tutorial, pretest, training and a posttest phase. Participants controlled a cartoon avatar (a fox) and were instructed to help the fox collect as many golden eggs as possible. The exact instructions are provided in the Appendix. The task began with a tutorial where the fox was placed in a lobby area with a single door in the middle. Participants were told that they could click on the door to switch between rooms. Once they did, the fox entered a room with eight chests, and participants were prompted to click on the chests to collect two golden eggs. When the participant clicked on a chest, the fox moved to inspect that chest. The chest was then opened to reveal whether an egg was inside. During the tutorial, all chests always contained eggs. Therefore, the subject’s first two choices were always rewarded. The total score (i.e., total eggs collected by participants) was always visible at the top of the screen.

Next, participants began the pretest, during which they once again started in the lobby area (**Figure 1B**), but were given a choice between two doors (blue and brown) on the sides of the room. The left or right placement of the two doors was counterbalanced across participants. The doors led to two visually distinct rooms (blue and brown, **Figures 1C,D**), which contained eight chests each arranged in a circular pattern. Both rooms were visually distinct from each other, and from the room encountered in the tutorial. For the next 4 min, participants were allowed to freely explore the virtual environment, switching between rooms and clicking on chests to acquire eggs. During the pretest, each chest had an initial 5% chance of containing reward. Throughout the task, whenever an egg was found in a particular chest, the chest’s subsequent chance of reward decreased to 0, and increased back to the maximum at increments of 1% every 4 s. Thus, repeatedly searching the same chest was not encouraged. Rather, the optimum strategy was to move around a room exploring different chests. Participants, however, were not told anything about reward contingencies and had to rely on trial-and-error. The amount of time spent in each of the rooms, the total number and order of chest clicks, and the total score was recorded. For each subject, the room (blue or brown) where that participant had spent more time during the pretest was defined as the “more preferred” room and the other as the “less preferred” room.

The pretest was followed by the training phase, which consisted of two parts (2 min each). At the start of each part of the training phase, the fox was placed in the lobby, but only one of the doors was available, forcing participants to enter one of the side rooms. Once they entered the room, they were locked in (**Figure 1E**) and had to remain there until the second part of training. The second part of training began in the same way, with the fox placed in the lobby and only the remaining door available. The less preferred room during the pretest was assigned to be the rich room, meaning that each chest had an initial 80% chance of containing an egg. The other room was assigned to be the poor room, where each chest had an initial 5% chance of containing an egg. As in the pretest, once an egg was found in a chest, reward chance decreased to 0 and gradually increased back to initial levels at increments of 10% (for the rich room) or 1% for the poor room, every 4 s. Whether participants were locked in the rich or the poor room first was counterbalanced. Again, the order and number of chests clicked was recorded, along with the number of eggs obtained.

Finally, participants completed a posttest, which was identical to the pretest. The fox was placed in the lobby with both blue and brown rooms freely available. All chests had an initial 5% chance of containing an egg. Here, the first room entered by participants, and the time spent in each room (previously rich vs. previously poor) were recorded, along with the order and number of chests clicked and the number of eggs obtained. After the task, all participants completed a questionnaire (see the Appendix) about their knowledge of reward contingencies, whether or not they had a strategy, and their computer or video game experience.

## Results

### Questionnaires

The mean score on the IUS-12 was 32.25 (*SD* = 8.58, range = 14–57). For all analyses, subjects were split into high or low IU groups based on the sample median of 32, with 37 participants (25 female) classed as low, and 39 (25 female) classed as high. The high and low IU groups did not differ in gender distribution, χ^2^(1) = 0.101, *p* = 0.750, or age, *t*(74) = 0.284, *p* = 0.778.

In the post-task questionnaire, in response to “Did you think that one of the rooms had more eggs in it?” 78.9% of participants responded “yes,” χ^2^(1) = 25.5, *p* < 0.001. Out of those who said “yes,” 90% also correctly identified the rich room, χ^2^(1) = 38.4, *p* < 0.001. Thus, most participants were explicitly aware of which room was more rewarding. Finally, 64.5% of the participants reported they had previously played computer or video games, χ^2^(1) = 6.4, *p* = 0.012, and 61.8% reported they had followed a specific strategy while searching for eggs, χ^2^(1) = 4.3, *p* = 0.039. Among the strategies mentioned were going in circles or zig zags and checking all of the chests once then switching rooms.

### Conditioned Place Preference Task

**FIGURE 2:**
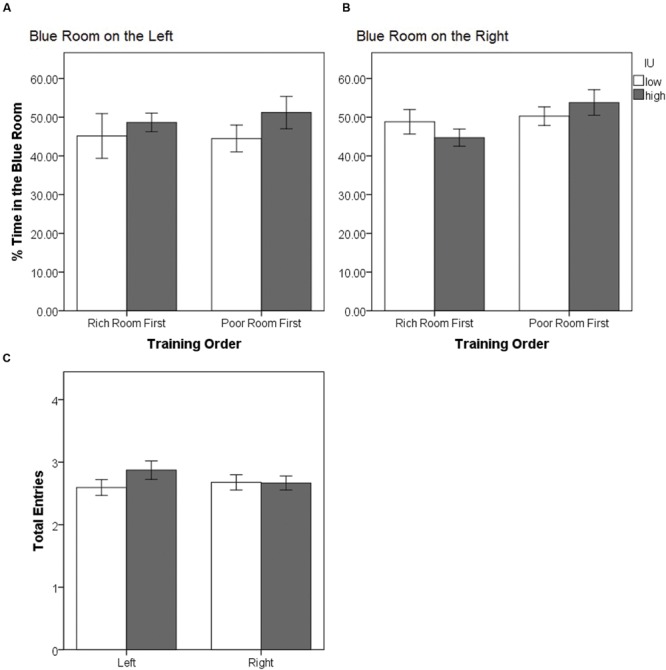
**(A,B)** Mean percent time spent in the blue room during the pretest as a function of training order and IU. There were no significant differences confirming that initially, all groups had no strong preference and divided their time equally between the blue and brown rooms. This was irrespective of which room was on the right or on the left, and whether the less-preferred room (assigned to be the rich room) was experienced first or second during training. **(C)** Average total number of entries into the left or right room during the pretest as a function of IU. All participants, irrespective of IU, made a similar number of visits to each room. Error bars represent ± SEM.

Since participants were assigned to one of four conditions to counterbalance which context (blue or brown) was on the left or right in the lobby, and whether the rich or the poor room was experienced first during training, we first examined whether this led to an initial preference bias as a function of IU. The mean percent of the time participants spent in the blue room during the pretest was computed as total time spent in the blue side room divided by sum of the total time spent in the blue and brown rooms (**Figures 2A,B**). A 2 (blue on left vs. right) × 2 (rich room first vs. second) × 2 (IU high vs. low) between-subjects ANOVA on the percent time spent in the blue room during the pretest confirmed that there were no significant main effects (all *F* < 1.720, all *p* > 0.19) or interactions (all *F* < 1.320, all *p* > 0.25). Thus, on average, participants tended to divide their time equally, spending approximately 50% of the time in each of the side rooms, eliminating initial bias as a potential explanation of the results in subsequent analyses. The average total number of entries made into each room by high and low IU participants was also examined (**Figure 2C**). A 2 (left vs. right room) × 2 (IU high vs. low) mixed-model ANOVA confirmed there were no significant main effects (both *F* < 0.900 and *p* > 0.34), and no significant interaction, *F*(1,74) = 1.618, *p* = 0.207. Thus, both groups of participants had enough time to make multiple visits to each room during the pretest.

**FIGURE 3:**
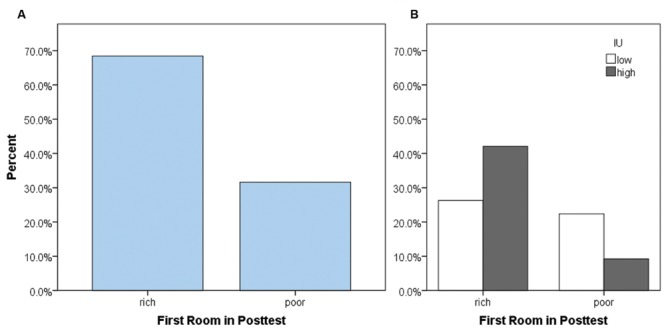
**Percent of total participants who chose to enter the previously rich or previously poor room first, at the start of the posttest. (A)** While most participants chose to enter the previously rich room first, surprisingly, about 30% went to the previously poor room first. **(B)** This behavior depended on IU. Participants with high IU were more likely to enter the previously rich room first in the posttest, and less likely to enter the previously poor room first, compared to participants with low IU.

As our primary analysis, we examined whether participants tended to enter the previously rich or the previously poor room first at the start of the posttest, i.e., whether they first entered the room paired with a high chance of reward (maximum 80%) or a low chance of reward (maximum 5%) during the training phase. It is important to note that during the posttest, both rooms were once again equivalent and paired with a low chance of reward (maximum 5%) as in the pretest. As expected, most participants entered the previously-rich room first, χ^2^(1) = 10.32, *p* = 0.001 (**Figure 3A**). However, surprisingly, approximately 30% of participants instead chose to enter the previously poor room. This could be due to differences in personality between participants, or a function of whether the last room experienced during the training phase was the rich or the poor room. To examine this possibility, we performed log-linear analysis – an extension of the chi-square test used for more than two categorical variables – on the total number of participants with factors of the first room entered during the posttest, the last room (rich or poor) experienced during the training phase, and IU (high or low). A non-hierarchical (forced-entry) method was used to enter factors into the model. The log-linear analysis produced a model that retained only the main effects and two-way interactions, and had a perfect fit to the data. The only significant two-way interaction was between the first room entered in the posttest and IU, $X_{p}^{2}$ (1) = 4.578, *p* = 0.032. The three-way interaction and the remaining two-way interactions (first room entered in posttest × last room in training and IU × last room in training) were not significant (all *X*^2^< 3.7, all *p*’s > 0.05). **Figure 3B** shows the percent of the total participants as a function of whether they entered the rich or the poor room first, and IU. Based on the odds ratio, participants who had high IU had 3.87 times higher odds of first going to the rich room in the posttest compared to participants who had low IU. Thus, participants with high IU tended to show greater CPP by going back to the previously rewarded context (i.e., followed a win-stay strategy) while those with low IU instead explored a different room (i.e., followed a win-shift strategy). The absence of other significant effects in the loglinear analysis suggests that this behavior was specifically a function of IU rather than other variables, such as which room participants had most recently been in during the prior training phase.

Similar analyses were performed to eliminate other possible confounds. IU and the first room entered in the posttest were always included in the model, while the third factor was whether or not participants reported they knew which room had more eggs (i.e., knew the rich room), had previous game experience or reported following a specific strategy. There was a significant three-way interaction between knowledge of the rich room, IU and the first room entered in the posttest, χ^2^(1) = 6.028, *p* = 0.014. To examine this interaction, a total of four Bonferroni-corrected two-sided Fisher’s exact tests (alpha adjusted to 0.05/4 = 0.0125) were performed with factors of IU and first room entered in the posttest. The first two tests were performed separately on individuals who reported they knew vs. did not know which room had more eggs. The result was significant only for individuals who reported they knew the rich room (*p* = 0.001 vs. *p* = 0.518). The second set of tests were performed on the subset of individuals who reported they knew the rich room, split by whether they also correctly identified that room. This confirmed a significant difference only for those who could identify the room (*p* = 0.001 vs. *p* = 0.467). Therefore, the interaction between IU and the first room entered during the posttest appears driven by participants who could explicitly identify the rich room. To avoid confusion, note that test statistics are not generated for Fisher’s exact test, therefore only *p*-values are reported. Finally, when game experience was examined, the model retained only the main effects and two-way interactions – the only significant two-way interaction was once again between IU and the first room entered in the posttest, $X_{p}^{2}$ (1) = 8.684, *p* = 0.003. Similarly this was the only significant two-way interaction when whether or not participants had a strategy was included as the third factor, $X_{p}^{2}$ (1) = 7.3, *p* = 0.007. Thus, neither game experience nor following a strategy were related to IU, or to which room participants entered first during the posttest. Across analyses, this depended on IU, and was also related to explicit knowledge of the rich room.

**FIGURE 4:**
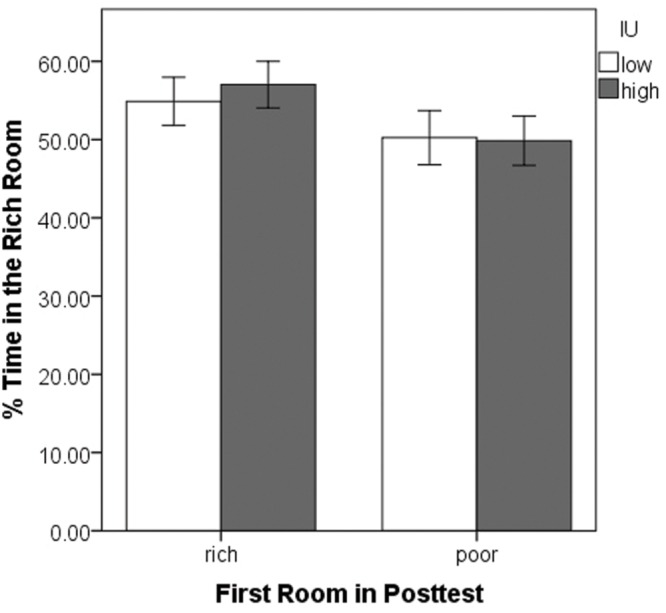
**Mean percent time spent in the previously rich room during the posttest as a function of the first room entered and IU.** There were no significant differences. Error bars represent ± SEM.

Having entered one room first in the posttest, we next examined whether participants tended to stay there, spending more time, overall, in that room. A 2 (rich vs. poor room entered first) × 2 (IU high vs. low) ANOVA was performed on the percent of the total time spent in the rich room during the posttest (**Figure 4**). This was calculated as total time in the rich room divided by total time in the rich plus the poor room. There were no significant differences (all *p*’s > 0.05). Thus, despite the initial preference to enter the previously rich room, most participants did not simply remain in the originally chosen room. Rather, across the whole posttest, participants tended to divide their time equally between the two rooms.

**FIGURE 5:**
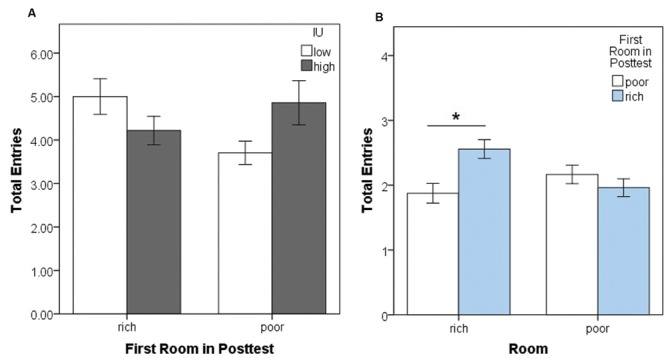
**Movement between rooms in the computer-based task. (A)** Mean total side room (blue and brown room) entries during the posttest as a function of the first room entered and IU. There was a significant interaction, however, *post hoc* independent samples *t*-tests fell short of corrected significance. **(B)** Mean total entries into the previously rich and -poor rooms during the posttest as a function of the first room entered. Significantly more re-entries were made into the previously rich room when that room was also the one first entered during the posttest. In contrast, total entries into the previously poor room were similar irrespective of which room was entered first. Error bars represent ± SEM. ^∗^ indicates significant difference, *p* < 0.01.

**FIGURE 6:**
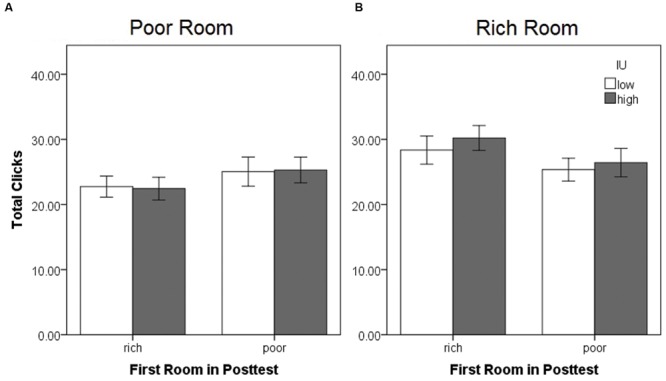
**Movement within each side room during the posttest expressed as the mean total chest clicks in the **(A)** previously poor and **(B)** previously rich room, as a function of the first room entered and IU.** There were no significant differences. Error bars represent ± SEM.

Next, we assessed locomotion in the posttest, first considering movement between rooms (**Figure 5**), then total chest clicks within each room (**Figure 6**). A mixed-model ANOVA was performed on total side room entries during the posttest with a within-subjects factor of the room entered (rich or poor), and between-subjects factors of the first room entered during the posttest (rich or poor) and IU (high or low). This yielded significant interactions between the first room entered and IU, *F*(1,72) = 4.71, *p* = 0.033, $\eta_{p}^{2}$ = 0.061, and between total entries into the rich or poor rooms and the first room entered, *F*(1,72) = 24.702, *p* < 0.001, $\eta_{p}^{2}$ = 0.255. There were no other significant interactions or main effects (all *p*’s > 0.05). *Post hoc* Bonferroni-corrected independent samples *t*-tests were conducted to further examine the significant interactions (alpha adjusted to 0.05/4 = 0.0125). The interaction between the first room entered and total room entries appeared to be driven by individuals with high IU making more entries into the poor room (**Figure 5A**), however, the test comparing entries into the previously poor room entries by high vs. low IU participants failed to reach corrected significance, *t*(22) = 2.18, *p* = 0.04. The interaction between total entries and the first room entered was due to participants who first entered the rich room tending to make more re-entries into that same room throughout the posttest, *t*(60.92) = 3.27, *p* = 0.002, *r* = 0.39 (**Figure 5B**). There was no significant difference in entries into the poor room as a function of which room was entered first during training. Overall, it is important to note that while there were some significant differences, effect sizes are small and the differences amounted to, on average, one or two additional room entries. More importantly, these data indicate participants remained active and continued to switch between rooms throughout the posttest.

**FIGURE 7:**
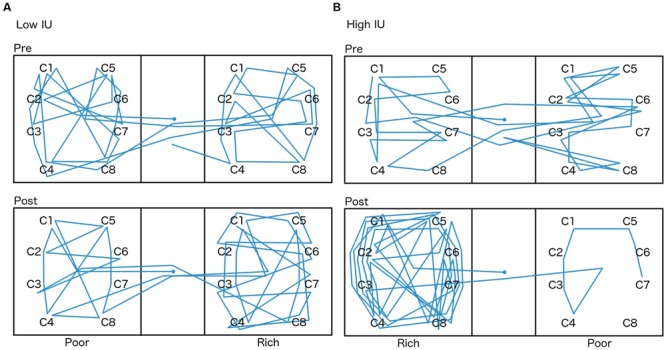
**Path taken by two participants chosen at random, one from the **(A)** low IU group, and one from the **(B)** high IU group, during the pretest and posttest.** The starting location, always in the lobby (center), is marked by a circle. The room each participant spent less time in during the pretest was assigned to be the rich room during training, and as described earlier, both side rooms had a maximum 5% chance of reward during the tests. The chests participants could search for eggs are marked with a “C.”

A mixed-model ANOVA was also performed on the total number of chest clicks (sum of the clicks on all eight chests) within each side room (**Figure 6**), with within-subjects factor of the room (rich or poor), and between-subjects factors of the first room entered (rich or poor) and IU (high or low). There were no significant differences (all *p* > 0.05). Sample graphs of the path taken by two individuals during the pretest and posttest, one from the low IU and one from the high IU groups (**Figure 7**), also indicate that participants remained motivated, continuing to switch rooms and check different reward locations, throughout the task. Note that while some individuals did show a strong preference for one room during the posttest (**Figure 7B**), on average, participants spent approximately equal amounts of time in both rooms, irrespective of IU. In contrast, as described earlier, individuals with high IU tended to visit the previously rich room first during the posttest.

**FIGURE 8:**
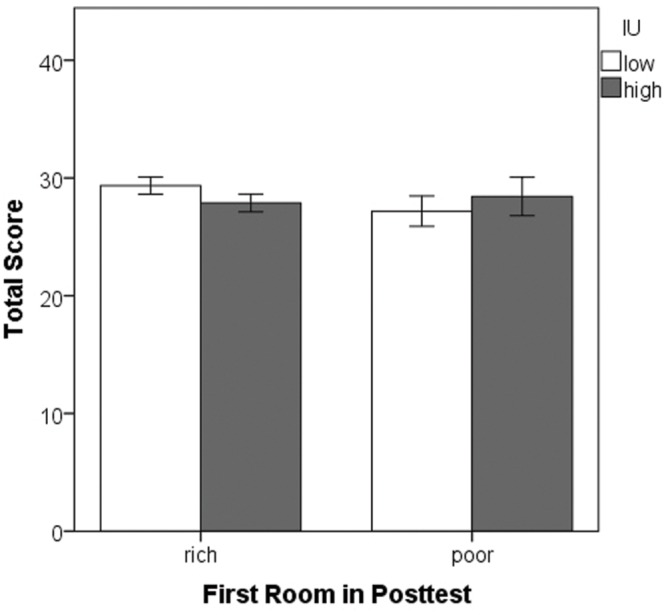
**Mean total score obtained in the task.** There were no significant differences. Note that the first two points all participants obtained during the tutorial do not count toward this total. Error bars represent ± SEM.

Finally, univariate ANOVA was performed on the total score (number of eggs collected) with between-subjects factors of the first room entered during the posttest and IU. There were no significant differences (all *p*’s > 0.05) indicating that on average, all participants obtained similar scores, irrespective of IU (**Figure 8**). Thus, differences in the amount of reward obtained during the training phase cannot account for the tendency of high IU individuals to choose to enter the previously rich room first during the posttest.

## Discussion

The current study found that individuals with low IU showed little bias to enter the previously rich room first, and instead entered both rooms at about the same rate. In contrast, those with high IU had a strong bias to enter the previously rich room first (i.e., increased win-stay). This interaction appeared to be driven by participants who could identify the previously rich room. These findings could not be explained by differences in initial room preference, by prior video or computer game experience or in the total reward obtained by participants. It was also not related to whether or not participants reported following a specific strategy in the task. There are at least two possible interpretations of this result – first, individuals with high IU may have selected the safer, more certain choice, by returning to the previously rewarded context. Second, this could also indicate an increased tendency to chase reward, consistent with previously observed behavior in heroin addicts in a probabilistic category learning task (Myers et al., 2016). In either case, this tendency may represent a pre-existing cognitive bias, possibly based on personality, which could promote decision-making processes that increase vulnerability to addiction. However, unlike the Myers et al. (2016) study, where the tendency to chase reward was expressed as exploration of new response options following expectancy violations (i.e., a lose-shift strategy), in the current task, participants chose the previously rewarded option (i.e., a win-stay strategy). This could be because, here, the response represents the first choice made in the posttest, precluding the influence of expectancy violations.

Another possible interpretation of this result, within an active inference framework (Friston et al., 2015), involves a change in the balance between pragmatic actions – actions that exploit previously rewarded strategies – and epistemic actions, which serve to discover new information that, long-term, may improve selection of pragmatic actions. According to this view, the value of an action is related to both its extrinsic value (i.e., expected reinforcement value) and its epistemic value (i.e., expected information gain). Here, actors seek out surprising outcomes, which will ultimately reduce uncertainty through information gain, and help construct a better internal model of the world. While short-term, this may require moving away from a goal (i.e., choosing to visit a previously less-rewarded location), the subsequent improved model would allow for better strategies to obtain a goal in the future (Friston et al., 2015). IU may involve a reduction in epistemic value in favor of increased extrinsic value, leading to behavior guided by preferences, i.e., prior beliefs about reinforcement contingencies. This interpretation is also consistent with the BI component of IU, which may paradoxically reduce information gain and therefore preclude resolving uncertainty in the long-term (Ladouceur et al., 1997; Krain et al., 2006).

Overall, we did not observe a CPP effect. That is, despite the fact that a majority of participants entered the previously rich room first, when considering behavior across the entire posttest period, participants did not spend a majority of the time in the previously rich room. This behavioral pattern may reflect a different strategy than simple CPP. Some individuals may have been foraging or choosing where and how to seek reward, much like animals search for food in the natural environment. Decision making in a foraging scenario would involve deciding between a limited number of options which have different probabilities of reward and amount of reward (Platt and Huettel, 2008). Foraging also includes some risk or cost in choosing to look elsewhere for food. This cost may take the form of energy expenditure to travel to some other location where more food may be available. Another cost is the amount of time that it would take to arrive at the other location. Cognitive decisions to forage involve several factors including the value of each option, an estimate of the average value in the environment, as well as the cost of leaving the current location to search elsewhere (Kolling et al., 2012). In the current task, there was very little cost of time or energy in moving from room to room. Therefore, this scenario did not include risk of energy expenditure or much time lost. Thus, the freedom to switch rooms without penalty would have made foraging behavior a viable strategy to possibly achieve more reward.

A tendency from the foraging literature that may have been expressed by our high IU group is known as the ambiguity effect, where when given a choice between two options, one in which the probabilities are known and one in which the probabilities are unknown, most avoid the option with no probability information (Camerer and Weber, 1992). This avoidance of a choice with unknown probability of outcomes could be an indication of IU. If high IU individuals knew which room was more rewarding, they may have tended to not shift their initial search to the other room, which in the past held less reward, but now may hold more. However, low IU persons exhibited a pattern of searching both rooms at similar rates. Low IU persons may have been more open to the risk of losing reward in the previously rich room if it was possible that more reward was available in the other room. Foraging has been tested in a computer environment (Goldstone and Ashpole, 2004), but the task involved a large number of participants interacting in real time in a virtual world. Based on a computational model, Goldstone and Ashpole (2004) suggested that some people tend to sample all locations with equal frequency while others tend to sample locations with greater rewards. Our current results would predict that these two tendencies may be found in two separate groups of individuals – those with lower IU would tend to sample all locations while those with higher IU would tend to sample locations of greater reward.

As noted earlier, individuals in the current study did not show an overall preference for the previously rich context. This could be because the current task differed in several ways from the CPP paradigms used in previous human and animal studies. In contrast to the work in animals, far fewer studies have attempted to examine CPP in humans. For example, in a study by Childs and de Wit (2009), humans received *d*-amphetamine or placebo in separate rooms. Participants reported higher liking for the drug-paired room. Molet et al. (2013) used a computer-based task where a distinct virtual environment was paired with either pleasant music or static noise. Analogous to animal studies, time spent in each context served as the dependent measure, and participants showed greater preference for the context paired with pleasant music. Finally, Astur et al. (2014) assessed preference for two distinct virtual rooms after one of them was paired with chocolate M&Ms. Similar to studies in animals, the participants spent more time in the chocolate-paired room, but only if they were food deprived.

Thus, similar to studies in animals, most human studies of CPP have employed either natural rewards (e.g., food, water) or drugs of abuse, while studies of economic decision making have used monetary gains. Nonetheless, most participants in the current study remained motivated throughout the task, despite only receiving golden eggs, as indicated by reliable movement and egg collection. The difference in the type of reinforcer, however, remains a possible explanation for the lack of overall preference in the current study, although note that Molet et al. (2013) were able to observe CPP to music. Similar to Childs and de Wit (2009), most participants in the current study reported that they knew which room was more rewarding, and were able to correctly identify that room. Despite this, there was no overall preference, and approximately 30% of participants first chose to enter the poor room during the posttest.

Another difference between this and other studies, which could account for the lack of overall preference, is in the duration of training. Animal studies typically involve multiple conditioning sessions, spread over several days, with training and testing on separate days. The study of Astur et al. (2014) in humans employed six 6-min sessions, with a 5 min break between each session, with training and testing on separate days. On the other hand, as in the current study, Molet et al. (2013) employed only two 2-min conditioning sessions but still observed a preference. However, in contrast to the current study, Molet et al. (2013) used an unbiased procedure where each context is paired with a stimulus, in counterbalanced order – there was no pretest. Here, a biased procedure was used where the least preferred room during the pretest was paired with reward. Additionally, Molet et al. (2013) did not restrict the duration of the preference test, while here both tests were 4 min long. This was to ensure participants remained motivated and to reduce frustration given that the chance of reward was, at most, 5% during each test. It is possible that participants did not have enough time to explore each of the available locations and become familiar with the task. This is unlikely, given that on average, participants were able to switch between rooms several times despite the time limit (as shown by the average total room entries). Still, the duration of the test may have precluded observing an overall preference, which could be examined in future studies.

Finally, unlike prior studies of economic decision making, participants did not receive more or less reward depending on their decisions to stay in each location. This was by design since differences in the reinforcement value between locations during the test would confound interpretation of any preference observed (i.e., such preference could be due to experiences during training and testing). In the future, the task could be modified to examine the preference between a poor room with potentially higher gains, but lower gain on average, and a rich room with lower but reliable gains. Similarly, the task could be modified to examine the effect of IU on both reward and punishment learning by introducing a chance to lose points when foraging in particular locations (e.g., to assess preference for a location associated with high risk and high reward). These alternatives may have a strong effect on preference, and alter the foraging strategy used by participants as a function of IU.

In both human and animal studies of CPP, reward is not contingent on operant responding. In the context of substance abuse, however, humans choose to start taking the drug and control the frequency of administration. Similar choices are involved in the context of foraging and economic decision making. Animal studies have typically used an operant conditioning paradigm to study these processes, where subjects learn to press a lever to self-administer drugs or obtain other rewards (Balster and Lukas, 1985; Bardo and Bevins, 2000). Most standard self-administration studies, however, do not consider the role of contextual cues. Thus, the current task combined the two approaches in order to examine both contextual conditioning, and placed reward under the control of participants. In doing so, the task likely also taps into different mechanisms compared to traditional CPP paradigms. For example, behaviorally, the magnitude of rodent CPP is often dissociated from the rate of self-administration (Bardo et al., 1999). The two paradigms also appear to engage different neural substrates. For example, pretreatment with D2 dopamine receptor antagonists has no effect on CPP to cocaine (Cervo and Samanin, 1995), but attenuates self-administration (Caine and Koob, 1994), suggesting that dopaminergic neurotransmission may only be involved in the primary reinforcing effects of cocaine, but not the secondary reinforcing properties acquired by contextual stimuli paired with cocaine (Bardo and Bevins, 2000). Finally, the ability of drugs of abuse to activate the mesolimbic dopamine system is also contingent on whether drug administration is under the operant control of the animal (Di Ciano et al., 1998).

Although the current task was probabilistic in that reward was not always guaranteed, the contrast between the two rooms during conditioning (5 vs. 80% chance of reward) should have been immediately apparent. When the rich room reverted to 5% chance of reward during the posttest, this may have led to rapid extinction, in particular since reward was under operant control, precluding observing a preference using time spent in the previously rich context as the dependent measure. While rodent CPP studies have used a 0 vs. 100% contrast, reward was not under operant control like it was in the current study. Regardless, the lack of an overall preference could also suggest that the effect of IU is not very strong in reality. Still, it may be possible to amplify this effect by increasing uncertainty (e.g., for example if the contrast is between 20 and 80% chance of reward). The number of chests in each context may have also played a role – the number was small enough to allow participants to explore all of the chests in one location before moving on and doing the same in the other. Thus, increasing the number of chests could influence how long participants choose to stay in one room, which could in turn impact overall preference.

In summary, we found a tendency for individuals who had high intolerance for uncertainty to first enter the previously rich reward room while individuals who had low intolerance showed no such bias, and first entered either of the rooms at equal rates. This initial decision may have been influenced by foraging strategies in addition to CPP. The results of the current study suggest that IU may have broader implications beyond the realm of anxiety, and is associated with changes in reward learning, even in healthy individuals. Studies are currently underway to examine the task in individuals undergoing treatment for opioid addiction. Given the relationship between IU and anxiety, such work should also compare addicts with and without comorbid anxiety disorders. It remains unclear if IU is an independent risk factor for both types of disorders, or if it is specific to individuals with comorbid anxiety that may have led to drug use in the first place, possibly as a form of self-medication (Khantzian, 1985). The current CPP task could also be adapted to examine a foraging scenario for further study of the effects of personality on economic decision making. This possible foraging task should include multiple rooms that the participant could explore for possible rewards. The cost for moving to other rooms could involve greater time delay that would reduce overall opportunity to forage. Thus, future computer-based behavioral tasks involving economic decision making could be used to test an individual’s foraging behavior in the context of IU, as well as other personality factors, and could also be used to assess how personality affects the disorders such as substance abuse and anxiety.

## Author Contributions

MR, CM, KB, AM, and MA were involved in study design. MA collected the data. MR, MA, and CM analyzed the data. MR, CM, KB, AM, and MA wrote the manuscript.

## Conflict of Interest Statement

The authors declare that the research was conducted in the absence of any commercial or financial relationships that could be construed as a potential conflict of interest.

The contents do not necessarily represent the official views of the Department of Veterans Affairs, the United States Government, or any institution with which the authors are affiliated.
