# Supplementary material for: The Personality Trait of Intolerance to Uncertainty Affects Behavior in a Novel Computer-Based Conditioned Place Preference Task
Source: Front Psychol. 2016 Aug 9;7:1175. doi: 10.3389/fpsyg.2016.01175 (PMC4977360; doi:10.3389/fpsyg.2016.01175)
Supplement: Supplementary file 1 [file Appendix.DOCX]

# Appendix

## Task Instructions

Instructions and prompts displayed on the computer screen over the course of the computer-based conditioned place preference task.

The first screen seen by participants:

*Help Kit the Fox collect golden eggs. First, you’ll practice how to collect eggs.*

*When you’re ready to begin, click start.*

This starts the tutorial. The following is displayed at the bottom of the screen:

*Try moving between rooms. Click on the center door.*

After clicking on the door:

*Great! Now, click on the chests to search for eggs. Try to find 2 golden eggs.*

Whenever an egg is found:

*+1*

The score counter displayed at the top also increments. When 2 eggs are found, the following is displayed:

*Next, you’ll start the real game.*

*Try to collect as many eggs as you can before time runs out.*

*When you’re ready to begin, click start.*

This starts the pretest phase. When time in each phase runs out:

*End of round <round number>. Please wait…*

At the end of each phase, except for the posttest, the screen fades out and the avatar appears back in the lobby. At the end of the posttest, the following appears on the screen:

*Thank you for playing.*

*Please tell the experimenter you’re done.*

## Posttask Questionnaire

1. Do you play computer or video games? (a. very often, b. sometimes, c. rarely/not anymore, d. never)

If a, b or c, what type of games?

2. Briefly, what do you think was the purpose of the game you just played?

3. Did you think that one of the rooms had more eggs in it? (a. yes, b. no)

If yes, which one? (a. blue/stain glass, b. brown/red brick)

4. Did you follow a specific strategy when searching for eggs? (a. yes, b. no)

If yes, what was your strategy?
